# Hereditary Multiple Exostoses—A Review of the Molecular Background, Diagnostics, and Potential Therapeutic Strategies

**DOI:** 10.3389/fgene.2021.759129

**Published:** 2021-12-10

**Authors:** Ewelina Bukowska-Olech, Wiktoria Trzebiatowska, Wiktor Czech, Olga Drzymała, Piotr Frąk, Franciszek Klarowski, Piotr Kłusek, Anna Szwajkowska, Aleksander Jamsheer

**Affiliations:** ^1^ Department of Medical Genetics, Poznan University of Medical Sciences, Poznan, Poland; ^2^ Medical Student, Poznan University of Medical Sciences, Poznan, Poland; ^3^ Centers for Medical Genetics GENESIS, Poznan, Poland

**Keywords:** hereditary multiple exostoses (HME), multiple osteochondromas (MO), diaphyseal aclasis, *EXT1* gene, *EXT2* gene, HME molecular backround, HME diagnostics, HME therapeutic strategies

## Abstract

Hereditary multiple exostoses (HMEs) syndrome, also known as multiple osteochondromas, represents a rare and severe human skeletal disorder. The disease is characterized by multiple benign cartilage-capped bony outgrowths, termed exostoses or osteochondromas, that locate most commonly in the juxta-epiphyseal portions of long bones. Affected individuals usually complain of persistent pain caused by the pressure on neighboring tissues, disturbance of blood circulation, or rarely by spinal cord compression. However, the most severe complication of this condition is malignant transformation into chondrosarcoma, occurring in up to 3.9% of HMEs patients. The disease results mainly from heterozygous loss-of-function alterations in the *EXT1* or *EXT2* genes, encoding Golgi-associated glycosyltransferases, responsible for heparan sulfate biosynthesis. Some of the patients with HMEs do not carry pathogenic variants in those genes, hence the presence of somatic mutations, deep intronic variants, or another genes/loci is suggested. This review presents the systematic analysis of current cellular and molecular concepts of HMEs along with clinical characteristics, clinical and molecular diagnostic methods, differential diagnosis, and potential treatment options.

## Introduction

Hereditary multiple exostoses (HMEs) syndrome, also known as multiple osteochondromas, hereditary deforming chondrodysplasia, multiple cartilaginous exostoses, or diaphyseal aclasis, was first described in a French family by Alexis Boyer in 1814 (Hennekam, 1991). It is a rare orphan disease with unknown exact incidence due to asymptomatic individuals that remain undiagnosed (Stieber and Dormans, 2005). However, some researchers estimate that HMEs occur in one per 50,000 in the Western population and affect more often males reaching male to female ratio as high as 1.5 (Schmale et al., 1994; Bovée, 2008; Ryckx et al., 2013; Pacifici, 2017).

**FIGURE 1:**
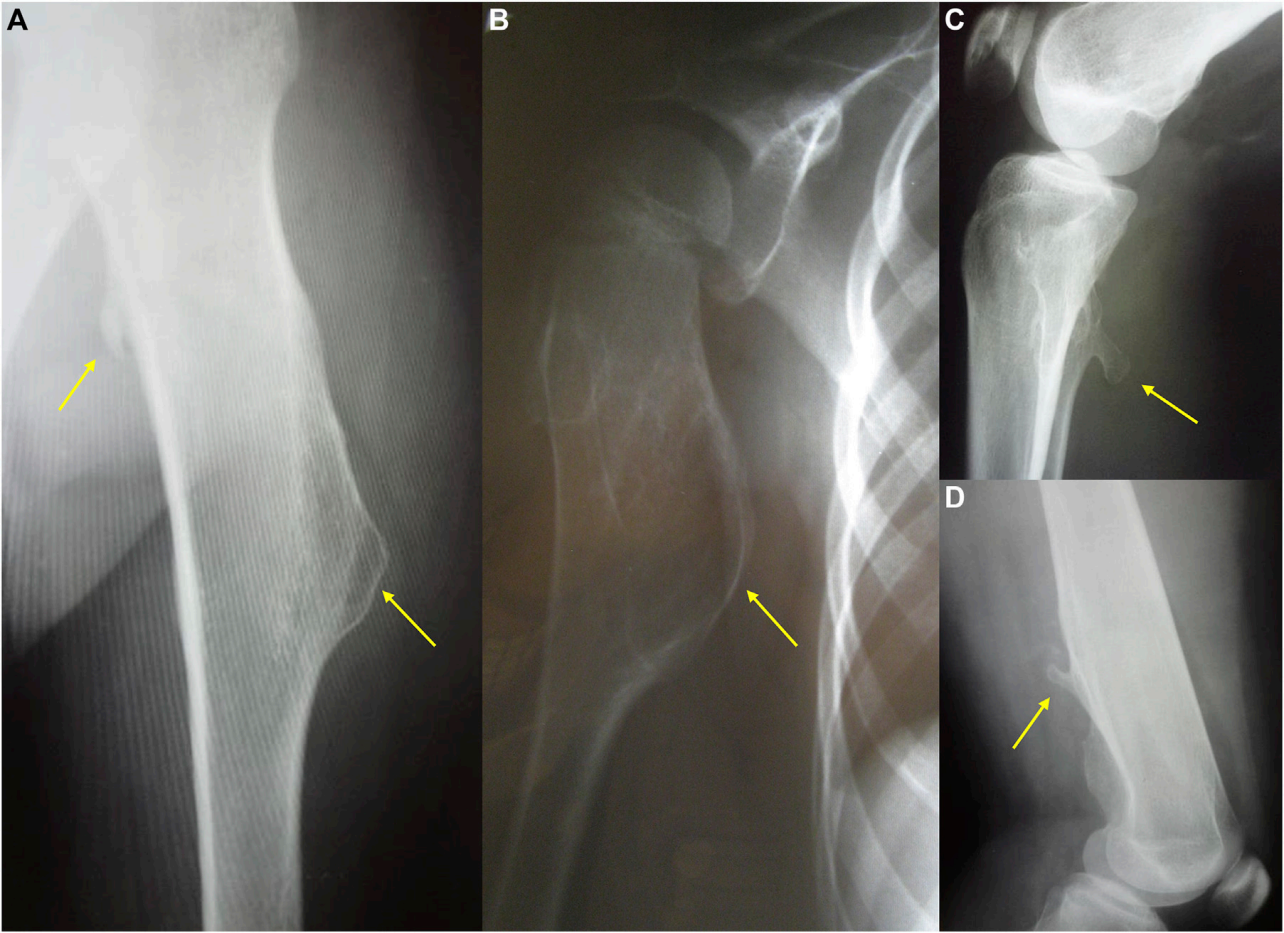
The radiography showing two different types of exostoses. Pictures **(a,b)** present sessile, mound shape osteochondromas, while **(c,d)** show pedunculated exostoses.

**FIGURE 2:**
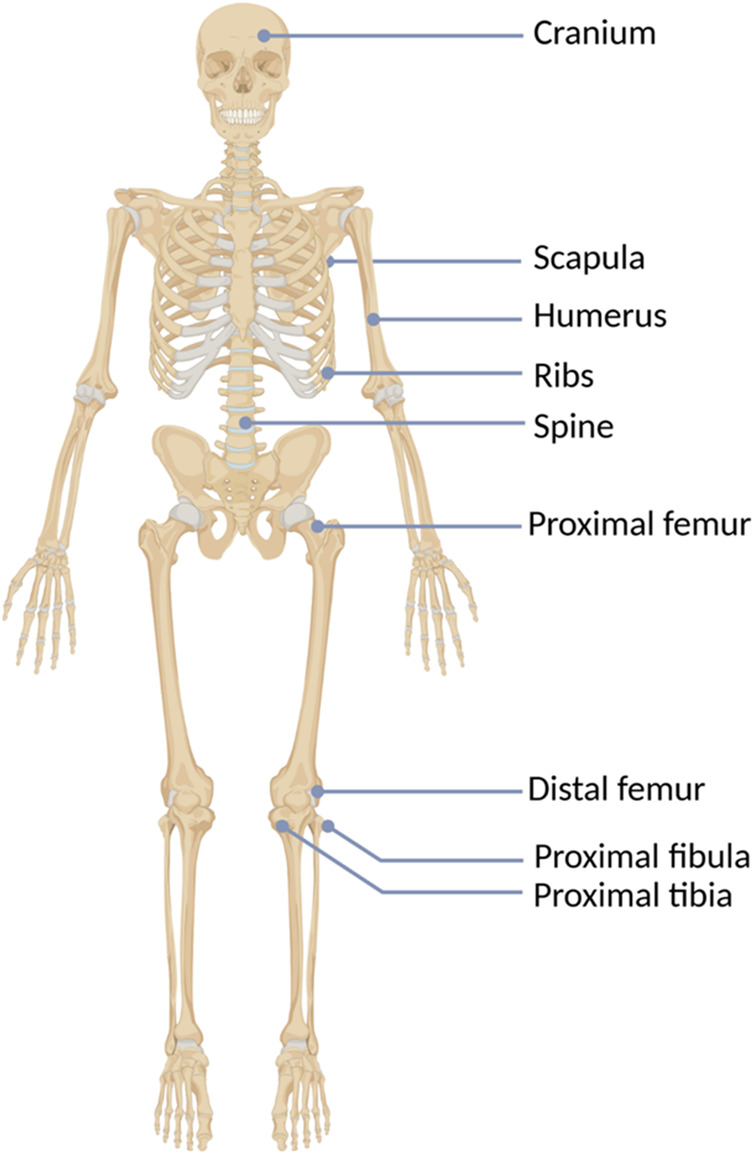
The hereditary multiple exostoses (HMEs) localization in the human skeleton.

HMEs develop in early childhood as benign multiple cartilage-capped bone tumors, i.e., osteochondromas, which ossify when skeletal growth is complete. The osteochondromas mainly involve long bone metaphyses and diaphyses, including ribs, but rarely also the scapula, vertebrae, pelvis, and sporadically the calvarial base (Figures 1, 2) (Stieber and Dormans, 2005; Sinha et al., 2017). Consequently, affected individuals may present a reduction of skeletal growth, short stature, bone deformities, scoliosis, premature osteoarthrosis, or compression of peripheral nerves (Wuyts et al., 1993; Vanhoenacker et al., 2001). However, the most severe complication of HME is a sporadic malignant transformation into chondrosarcoma (CHS), which risk depends on age, sex, genotype, and anatomical distribution of exostoses (Stieber and Dormans, 2005; Czajka and DiCaprio, 2015). One simple and valuable classification system, reflecting the severity of symptoms, was proposed by Mordenti et al. in 2013. It divides HMEs into three classes (I-III), depending on the presence or absence of the functional limitations and deformities. Additionally, each class includes two subclasses (A or B), which inform about the number of the affected body regions (Table 1) (Mordenti et al., 2013).

**TABLE 1:** Clinical hereditary multiple exostoses (HMEs) classification obtained *via* Switching Neural Networks approach, proposed by Mordenti et al. (2013).

Class	Subclass
I: deformities- no, functional limitations- no	A: ≤5 sites with exostoses
	B: >5 sites with exostoses
II: deformities- no, functional limitations- present	A: ≤5 sites with deformities
	B: >5 sites with deformities
III: deformities- present, functional limitations- present	A: 1 site with functional limitation
	B: >1 site with functional limitation

## Molecular Background

**FIGURE 3:**
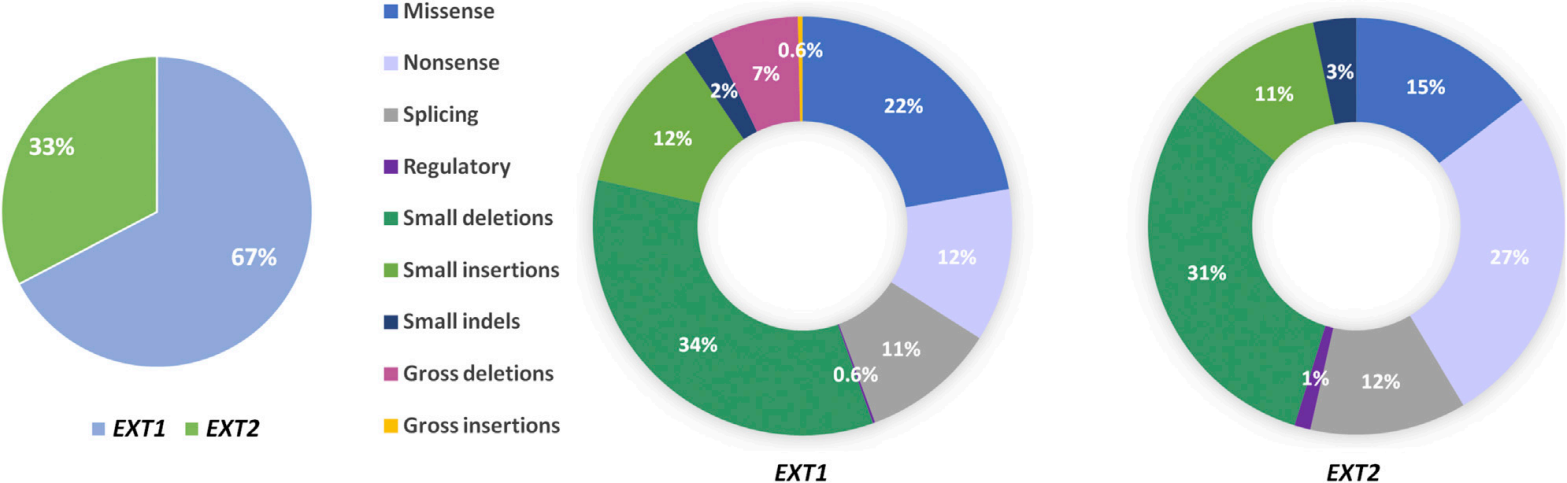
Pie charts showing the percentage of mutation types found in the *EXT1* and *EXT2* genes in patients affected with hereditary multiple exostoses (HMEs). Data were obtained from Human Gene Mutation Database (HGMD v.2021.1; accessed on 25^th^ of May).

**FIGURE 4:**
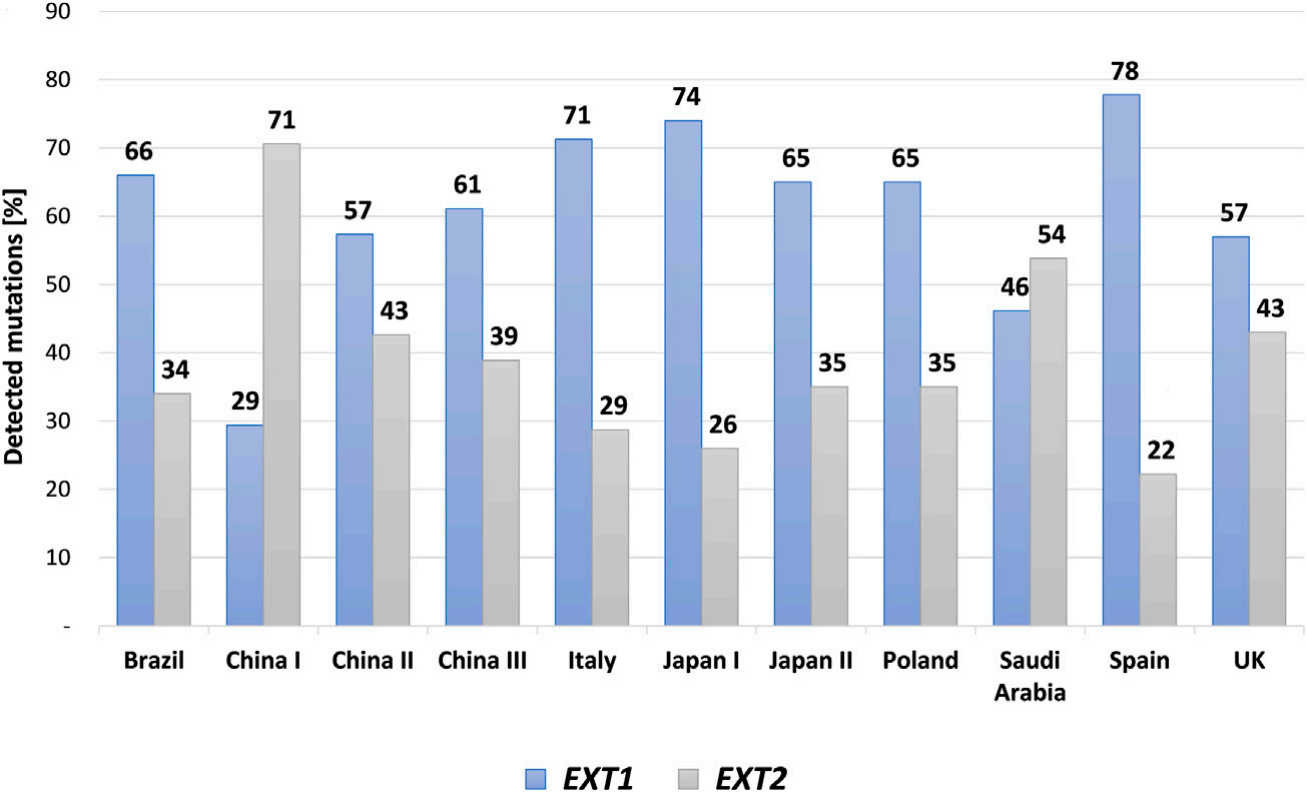
A bar graph showing mutational discrepancies in the *EXT1* and *EXT2* among different cohorts of patients affected with hereditary multiple exostoses (HMEs) and reported in the medical literature. The following cohorts were included: Brazilian, Chinese, Italian, Japanese, Polish, Saudi Arabic, Spanish and United Kingdom (UK) populations.

HME syndrome results from pathogenic variants located within the *EXT1* (8q24.11) and *EXT2* (11p11.2) genes that cause HME type 1 (MIM: 133700) and type 2 (MIM: 133701), respectively (Ahn et al., 1995; Wuyts et al., 1995; Stickens et al., 1996; Hecht et al., 1997). A detailed summary of all reported mutation types identified in each gene was shown in Figure 3. Interestingly, some discrepancies in the prevalence of *EXT1* and *EXT2* mutations have been shown among various populations of HME patients, including individuals of Asian, Caucasian, and Latin American origin. In most of the ethnicities, pathogenic variants occur more often within the *EXT1* gene (Figure 4). However, some exceptions were also reported in the medical literature such as results from Chinese HME patients’ screening that revealed the higher incidence of *EXT2* mutations (71%, 12/17) than *EXT1* (29%, 5/17) (Xu et al., 1999). Similarly, a recent report from Saudi Arabia revealed 54% (7/13) of mutations in the *EXT2* gene and 46% (6/13) in the *EXT1* gene (Al-Zayed et al., 2021). Interestingly, the subsequent two analyses performed among the Chinese put in question the previous results. Li et al. reported 57% of the (39/68) pathogenic variants in the *EXT1* gene and 43% (29/68) in the *EXT2* gene (Li et al., 2018). Wang et al. have also described a higher incidence of *EXT1* mutations, identified in 61% (11/18), whereas *EXT2* variants in 39% (7/18) of all cases (Wang et al., 2020). On the other hand, two independent studies performed in Japanese patients gave fairly consistent results. In the first analysis, variants in the *EXT1* gene accounted for 74% (17/23), whereas in *EXT2* for 26% (6/23) (Seki et al., 2001). The second study revealed that 65% (34/52) of all mutations were identified in *EXT1*, while 35% (18/52) were localized in *EXT2*. Interestingly, three families harbored variants in both genes (Ishimaru et al., 2016)*.* The study performed among Brazilian, Italian, Polish, Spanish, and United Kingdom HME cohorts suggested that pathogenic variants more often localize in the *EXT1* gene rather than in *EXT2* gene (Figure 4) (Lonie et al., 2006; Sarrión et al., 2013; Jamsheer et al., 2014; Santos et al., 2018; Fusco et al., 2019). Furthermore, data obtained from the Human Gene Mutation Database (HGMD), provide clear evidence that the number of pathogenic variants in *EXT1* is higher than causative alterations in *EXT2* (Figure 3).

Notably, in all reported studies, a significant proportion of patients remained molecularly undiagnosed. The percentage of cases without either *EXT1* or *EXT2* causative alteration ranged from 4% up to 33%, depending on the type of mutational analyses performed. Consequently, the presence of another yet unidentified causative gene for HME, provisionally termed *EXT3* and linked to chromosome 19p locus, was postulated (Le Merrer et al., 1994; Francannet et al., 2001). Second, mosaic mutations of the *EXT1* or *EXT2* genes may also account for HMEs in at least some of the patients negatively tested for germline mutations in both genes (Stieber and Dormans, 2005). One may also suspect the presence of deep intronic or regulatory variants in both *EXT1* and *EXT2* genes, which are undetectable using standard diagnostic methods, i.e., PCR followed by Sanger sequencing or targeted next-generation sequencing (NGS) methods, such as genes panel analylsis and whole-exome sequencing (WES).

## Cellular Background

Studies have proved that intracellular processes leading to the formation of osteochondromas depend on aberrant heparan sulfate (HS) biosynthesis. HS backbone formation is mediated by two glycosyltransferases encoded by the *EXT1* and *EXT2* genes (Majumdar, 1994; Busse-Wicher et al., 2014). HS is a linear polysaccharide belonging to the glycosaminoglycan (GAG) family, composed of repeated disaccharide units. HS chains may attach to “core protein”, forming one type of proteoglycans (PGs) called, in such a case, heparan sulfate proteoglycans (HSPGs). HSPGs are exposed on both the cellular membrane surface, i.e., pericellular and in an extracellular matrix (ECM), which plays a pivotal role in signal transduction of many molecules (Lopes et al., 2006). Thus, HSPGs and other PGs-GAGs are involved in various physiological and pathophysiological processes, such as receptor signaling, growth factor activation, cellular proliferation and differentiation, angiogenesis, and tumor metastasis (Trebicz-Geffen et al., 2008; Li and Kusche-Gullberg, 2016).

Glycosyltransferases encoded by the *EXT1* and *EXT2* genes are responsible for the biosynthesis of HSPG in the Golgi apparatus, where they compose a part of HS polymerase complex (Jones, 2011; Mikami and Kitagawa, 2013). In the following step, mature HPSGs are transported to the ECM and cell surface. Cells of the patients with HME have only one functional copy of either *EXT1* or *EXT2*, being more prone to lose their ability of HS synthesis compared to cells of unaffected individuals. In the case of a second-hit somatic mutation, and thereby random inactivation of a second gene copy, the cells cease to synthesize functional HS. Therefore, mutations in the *EXT1* and *EXT2* genes cause critical impairment of the HS chain synthesis and elongation, which manifests with the low levels of the pericellular and extracellular HS (Alvarez et al., 2006). It has also been suggested that reduction of extracellular HS impairs chondrocytes growth and differentiation factor, i.e., Indian hedgehog (IHH) or other factors involved in the bone formation such as fibroblast growth factors (FGFs), bone morphogenetic proteins (BMPs), WNT-proteins or parathyroid hormone-related proteins (PTHrPS) (Yoon et al., 2006; Jones et al., 2014; Billings and Pacifici, 2015; Mundy et al., 2016).

**FIGURE 5:**
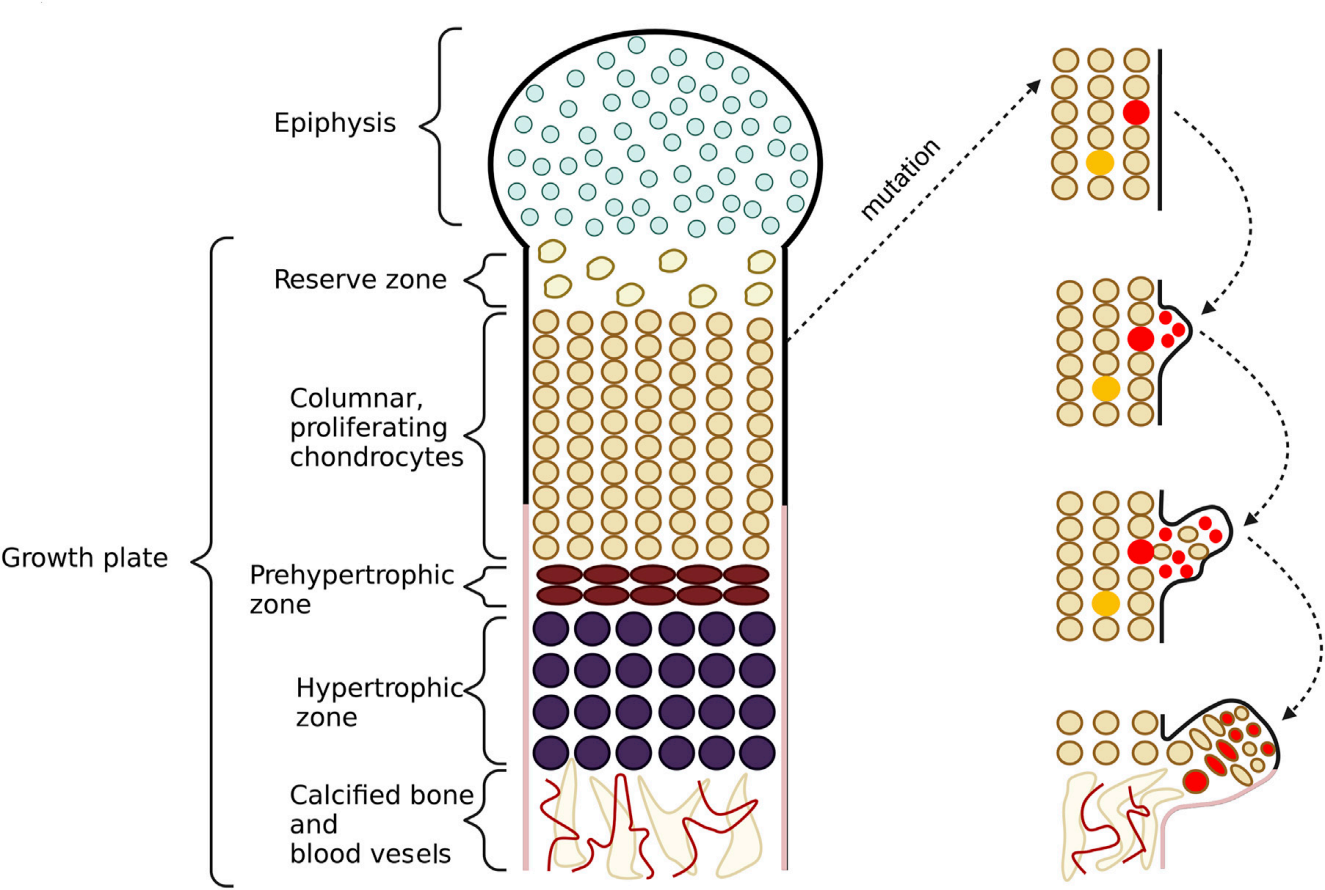
This figure shows the schematic formation of exostoses. In the growth plate, one can observe a proliferation zone of chondrocytes responsible for bone elongation. During this process, cells from the upper layers proliferate, while cells from the lower layers undergo apoptosis and then calcify. As a result of mutations in the *EXT1* and *EXT2* genes, chondrocytes with shortened heparan sulfate (HS) chains appear in this area. If the cell is located in the middle column, it can be rescued by the neighboring cells whith normal length HS chains. In this case, exostoses do not form. Conversely, if the cell is present in the outer columns, it escapes from a normal differentiation process. The cell loses its polarity but still keeps its proliferation potential. Then, the cell starts to translocate to lower layers of chondrocytes and grabs some non-mutated cells with it too. In the last stage, the cells form exostoses.

Consequently, abnormalities in bone growth occur as the complete lack of HS or shortened HS chains impair chondrocytes differentiation and proliferation processes. Some perichondrial progenitor cells of chondrocytes, located within the growth plate, change their proliferation direction due to the loss of their polarity, forming exostoses. Chondrocytes in the elongation region group into vertical columns, and only cells located in the externals columns can form tumors. Cells placed inside the middle columns probably use regular length HS from vicinal chondrocytes. Thus not all cells with shortened HS chains give rise to bone tumors (Jones, 2011). Pathological bone formations are usually found in long bones but rarely also in the flat ones (Alvarez et al., 2006). The schematic formation of HME was shown in Figure 5.

## Malignant Transformation

Multiple cartilage-capped exostoses in the course of HMEs develop in childhood and ossify when skeletal growth is complete. The bony outgrowths are benign tumors, although they can transform into malignant CHS in 3.9% of patients (Jones et al., 2014; Pacifici, 2017; Fei et al., 2018). CHS transformation usually occurs before the age of 40 years, and in 87% of cases involves the appendicular skeleton with the most frequent localizations involving the pelvis followed by the scapula, proximal femur or spine, and the ribs. CHS is usually low-grade, but the main limitation for radical surgical excision may be the close location of the tumor to the principal neurovascular bundles. Several studies have suggested that individuals carrying *EXT1* mutations are statistically at 1.5–2 times greater risk of malignant transformation than those harboring the *EXT2* pathogenic variants (Fei et al., 2018).

## Clinical Manifestation

HMEs are rarely detected at birth. Usually, they gradually develop during childhood and adolescence and cease to grow once the epiphyseal plates become mature (Vanhoenacker et al., 2001; Pedrini et al., 2011). The average number of exostoses per patient is 15–18, however, it varies considerably among affected individuals and ranges from 2 to 172 osteochondromas (Bovée, 2008; Clement and Porter, 2014). HMEs may develop in bones formed in the process of endochondral ossification, most frequently long bones. One of the most commonly involved body regions comprises the knee area, with exostoses occurring predominantly in the distal femur (70%), proximal tibia (71%), and proximal fibula (27%). Although HMEs predominantly locate in the skeleton of the lower extremities, other frequently affected bones include humerus (50%), ulna and radius (30%) (Schmale et al., 1994). Less common locations involve hands, ribs, scapulae, vertebrae, and pelvis (Figure 2) (Guo et al., 2014).

The clinical severity of the disease depends on the total number of exostoses as well as their size and shape. Osteochondromas typically project away from the epiphysis and may be pedunculated or sessile. The osseous tuber is called pedunculated when it is slender and has a narrow stalk. This form tends to irritate the surrounding tissues, leading to local trauma. The sessile tuber has semicircular or mound shape and attaches to the bone with a broader base (Figure 1). Some authors suggest that pedunculated morphology and the massive exostoses with greater cortical involvement associate with notable shortening of an involved bone (Stieber and Dormans, 2005).

The exostoses in their non-transformed form are benign lesions that can remain almost asymptomatic until they are detected by palpation. Unfortunately, the majority of patients are symptomatic, as over 80% of them experience pain, which is the most common disease complaint (Darilek et al., 2005). Pain may arise secondary to the irritation of structures adjacent to bone tubers, f. e. muscles, tendons, blood vessels, peripheral nerves, spinal cord, but also from joint malalignment or bursitis. Exostoses are usually located in the proximity of joints, limiting their range of motion, restricting their flexion or rotation (Jäger et al., 2007; Clement and Porter, 2014). Due to an outgrowth of the epiphyseal plate, osteochondroma may interfere with skeletal development and give rise to limb deformities. Disproportionate bone shortening can result in joint malalignment, bowing, subluxation, or dislocation of adjacent bones. These deformities include Madelung-type deformities of the wrist, hand involvement (including brachydactyly, pseudomallet fingers, angular deformity), coxa valga, acetabular dysplasia, genu valgum, and valgus angulation of ankle joint (Vanhoenacker et al., 2001; Stieber and Dormans, 2005; Beltrami et al., 2016). Limb length discrepancy is frequent and, in many cases, requires surgical interventions. Apart from the local effects of osteochondromas, a widespread influence of HME on skeletal system development was also suggested. Matsumoto et al. showed that more than half of HME patients present with mild to moderate scoliosis, independent of lower limb deformity or the number of osteochondromas (Matsumoto et al., 2015). Another systemic influence associated with HME is short stature. The skeletal age in younger children with HME is retarded in reference to their metrical age, while for adolescents, it is accelerated, suggesting an earlier closure of the growth plates (Staal et al., 2015). In general, most adult patients’ height is below average but within a normal range (Goud et al., 2012).

Rare and atypical complications of HME may also occur. First, thoracic exostoses have been reported to give rise to pneumothorax, diaphragmatic rupture, hemothorax, coronary artery compression, and severe chest pain (Wuyts et al., 1993; Vanhoenacker et al., 2001; Cowles et al., 2005; Ravindran et al., 2015). Second, cervical osteochondromas have been found to cause neurogenic and vascular thoracic outlet syndrome as well as dysphagia (Gulati et al., 2013; Abdolrazaghi et al., 2018). Finally, a malignant transformation can develop in 3.9% of all affected patients, being the most severe complication of HME (Czajka and DiCaprio, 2015; Jurik, 2020). Malignant transformation occurs more frequently in particular anatomical regions such as the pelvis, scapula, and proximal part of the femur. Tumors arising from endochondral elements of the cranium and defects of the cranial base, especially clivus, are also common but often overlooked in the diagnostic screening (Sinha et al., 2017).

## Diagnosis and Differential Diagnosis

The first symptoms of HME are frequently noticed by the parents when the patient is 5–7 years old and recognized because of disabilities in gross and minor motor skills or local pain, which develops due to osteochondromas’ pressure on the neighboring tissues (Czajka and DiCaprio, 2015; D’Arienzo et al., 2019). The clinical HME diagnosis is based on imaging tests such as X-rays, CT or MRI. The disease is recognized when at least two osteochondromas are identified in an individual, and other possible diagnoses are simultaneously excluded (D’Arienzo et al., 2019). The lesions mainly localize at the surface of the bone and in the metaphyseal regions, and to be considered bona fide osteochondromas, they need to maintain contact with the underlying parent bone cortex and the medullary canal (Murphey et al., 2000; Alabdullrahman and Byerly, 2021). As HMEs may clinically overlap with several other diseases, the differential diagnosis presented in Table 2 should be considered.

**TABLE 2:** A comparison of hereditary multiple exostoses (HMEs) and other disorders presenting overlapping clinical features (Maas et al., 1993; Pignolo et al., 2013; McFarlane et al., 2016; Beyens et al., 2019; Jurik, 2020; Suster et al., 2020; Trajkova et al., 2020; Charifa et al., 2021).

Disease	Similarities*	Differences*
Metachondromatosis	multiple exostoses and enchondromas	pathogenic variants in the *PTPN11* gene in some individuals
Ollier disease and Maffucci syndrome	multiple enchondromas	somatic mosaic pathogenic variants in the *IDH1, IDH2*; *PTH1R* genes in some individuals
Langer–Giedion syndrome (Trichorhinophalangeal syndrome)	multiple exostoses, growth retardation, *EXT1* loss of function mutations	distinctive facial features, ectodermal features, intellectual disability in some individuals, partial interstitial deletion of the chromosome 8 (8q24), including the *EXT1* and *TRPS1* gene
Potocki–Shaffer syndrome	multiple exostoses *EXT2* loss of function mutations	biparietal foramina, neurodevelopmental delay, intellectual disability, facial dysmorphism, partial interstitial deletion of the chromosome 11 (11p11.2), including the *EXT2*, *ALX4* and *PHF21A* genes
Fibrodysplasia ossificans progressiva	numerous bone tumors	foci of ectopic ossification, ossifying changes of muscular and other tissues, hypoplastic or absent halluces, pathogenic variants in the *ACVR1* gene
Menkes disease (Occipital horn syndrome)	bony exostoses	cutis laxa and bladder diverticula, pathogenic variants in the *ATP7A* gene
Gardner syndrome	multiple exostoses	numerous adenomatous polyps in gastrointestinal tract, dental anomalies, skin changes, malignant tumors in various locations, variants in the *APC* gene

*to HMEs.

Radiological imaging is recommended and usually becomes the first step of the diagnostic process. However, it is sufficient only when HMEs localize within long bones (D’Arienzo et al., 2019). Upon radiographic imaging, the characteristic sign of exostoses includes a marked borderline between the tumor and the healthy bone. In addition, CT and MRI more accurately identify skeletal changes in the abdominal cavity and the pelvis due to limited accessibility of these locations to standard X-ray imaging (Kok et al., 2013; Beltrami et al., 2016). Moreover, MRI allows for detailed visualization of bone structures, including distinction between benign and malignant tumors. Even if new tumors no longer occur after the inhibition of bone development at about 20–25 years of age, there is still a possibility of malignant transformation into CHS. Therefore, a whole-body MRI scan should be implemented as screening for early detection of CHS in patients with HME, regardless their age (Kok et al., 2013; Jurik et al., 2020). Herget et al. f. e. propose that MRI should be performed once a year (Herget et al., 2013). Also, preoperative MRI is recommended to reduce postoperative complications resulting from unidentified intraspinal exostoses (Vu et al., 2020).

Other under-estimated and rarely implemented methods in HME diagnosis include single positron emission computer scan (SPECT-CT) and specific biomarkers analysis. Although SPECT-CT is rarely performed due to its low availability and high cost, Van den Wyngaert was fairly enthusiastic about the possibilities of this method regarding its sensitivity and specificity compared to CT imaging or bone scintigraphy (Van den Wyngaert et al., 2020). Likewise, Wang emphasizes that SPECT-CT has an excellent diagnostic value as the method of bone disease diagnosis (Wang et al., 2018).

In addition to imaging methods, the highest specificity for HME diagnosis confers genetic testing, which can identify the disease even before the development of the first symptoms, giving a possibility to plan a long-term follow-up (Medek et al., 2017). Genetic counseling is particularly recommended for adults who have a child with HME to estimate the risk of the disease in the future offspring as well as adults with HME who plan a pregnancy. Still, molecular testing seems indispensable in the postnatal screening of children born to affected individuals and their relatives at risk (Liang et al., 2020). As only two genes, i.e., *EXT1* or *EXT2*, have been linked with HME thus far, they are routinely screened to detect point mutations, intragenic deletions or duplications, or rarely, more complex rearrangements. Methods that have been implemented for causative mutation screening involve PCR followed by Sanger sequencing, MLPA (MRC Holland kits), quantitative PCR, or, if available, next-generation sequencing-based methods.

Finally, Anower-e-Khuda et al. revealed the potential biochemical markers available for testing in individuals’ blood, such as HS/chondroitin sulfate (CS) ratio or HS level. Both HS/CS ratio and HS level decrease in the course of HME. The HS/CS ratios of HMEs patients were almost half of those identified in healthy controls (Anower-E-Khuda et al., 2013).

## Treatment and Novel Therapeutic Strategies

Current methods of HME treatment bases on surgical removal of exostoses, especially those symptomatic or causing damage and irritation to the local structures. In the case of asymptomatic osteochondromas, no therapy is implemented. Surgical treatment intends to relieve chronic pain reported by most patients and prevent them from skeletal deformities, which often include growth asymmetry, resulting f. e. in limb length discrepancy. Moreover, it is performed to restore the motion of joints, improve circulation hampered by vessel compression, or for cosmetic purposes (Stieber and Dormans, 2005; Bovée, 2008). However, complete excision of osteochondromas in some regions may not be possible (Phan et al., 2018). Additionally, as shown in the study published in 2012, patients with HME present a lower Health-Related Quality of Life factor in comparison with the control group (Chhina et al., 2012). For these reasons and because of everyday life discomfort experienced by many patients, new potential treatments need to be investigated (Sabir and Cole, 2019).

In HME, at the cellular level, the biosynthesis of heparan sulfate is affected, which is crucial for the proper functioning of many metabolic pathways. It mainly involves the FGF, BMP, hedgehog, and retinoid signaling pathways, out of which any can turn out to be a potential treatment target (Huegel et al., 2013; Pacifici, 2018). Extensive research has been done on the most promising potential drug, i.e., palovarotene (PVO), a selective agonist of retinoic acid receptor *γ*, present in chondrocytes and mesenchymal precursor cells. Its mechanism of action is connected with blocking chondrogenesis and cartilage development at the level of retinoid signaling. Previous studies revealed that chondrogenesis and growth plate functioning are enhanced by silencing of retinoid signaling (Hoffman et al., 2003). Furthermore, it involves the repressive mechanism of retinoic acid receptors (RAR), which—when unliganded with retinoic acid—exert repressor function to the target genes in the nucleus (Weston et al., 2002; Shimono et al., 2011; Pacifici, 2018). Studies performed *in vitro* as well as in mouse and rat models have shown that active retinoids can block the process by enhancing retinoid signaling, leading to suppression of chondrogenesis (Wang et al., 2014; Inubushi et al., 2018). In 2018, *Clementia Pharmaceuticals Inc.* (now incorporated by *Ipsen* group) launched a phase two, randomized, double-blind, placebo-controlled clinical trial on PVO efficacy and safety in patients with HME (ClinicalTrials.gov Identifier: NCT03442985). However, the FDA partially suspended the study in 2019 due to the reports about early growth plate closure in children treated with PVO in another clinical trial regarding fibrodysplasia ossificans progressiva (FOP). The study of PVO treatment in patients with HME was eventually terminated in October 2020 to analyze collected data. No reports describing the results and treatment efficacy have been released yet.

Other potential and promising treatment targets are presented in Table 3. They include BMP and Hedgehog signaling pathways or an enzyme heparanase (Yoon et al., 2006; Bovée et al., 2010; Heuzé et al., 2014; Huegel et al., 2015; Mundy et al., 2016; Sinha et al., 2017; Inubushi et al., 2018; Pacifici, 2018).

**TABLE 3:** The presentation of potential treatment targets in hereditary multiple exostoses (HME) (Yoon et al., 2006; Bovée et al., 2010; Heuzé et al., 2014; Huegel et al., 2015; Mundy et al., 2016; Sinha et al., 2017; Inubushi et al., 2018; Pacifici, 2018).

Potential treatment target	Clarification
BMP signaling pathway	BMP (bone morphogenetic protein) signaling plays an essential role in skeletal development by regulating chondrocyte proliferation and differentiation. Inubishi et al. showed the connection between increased BMP signaling and osteochondromagenesis. In addition, they suggested that treatment with BMP inhibitor may be effective in HME patients, which was proved by the promising results of their study conducted on mouse models. Administration of BMP inhibitor LDN-193189 presented a suppressive effect on osteochondroma formation. Another study in mice brought similar results
Hedgehog signaling pathway	The hedgehog signaling pathway regulates the proliferation of growth plate chondrocytes. In this regard, the response to the potential treatment of HME with Hedgehog Signalling Antagonist (HhAntag) has been investigated. Studies *in vitro* not only demonstrated an effective block in chondrogenesis but in addition, inhibition in the BMP signaling pathway has been observed, showing the complexity of mechanisms involved in these processes
Heparanase	Heparanase, an enzyme that cleaves the heparan sulfate (HS) chains and stimulates chondrogenesis, is physiologically found only in the hypertrophic zone and perichondrium. Its wider distribution and increased activity possibly play a role in the development of osteochondromas. An *in vitro* study showed that a heparanase antagonist SST0001 successfully inhibited chondrogenesis. It suggests that further investigation may be profitable

## Conclusion

HME is a rare pediatric skeletal disease in which benign tumors next to bone growth plates develop. The disease may severely affect the quality of patients’ life due to motion impairments, skeletal deformations, chronic pain, or growth retardation and possibility of malignant transformation of exostoses. Despite the studies on potential causal therapies, the only available treatment options are surgical removal of the most symptomatic tumors, correction of skeletal defects, and analgesic interventions. Although the treatment strategies are limited, the precise diagnosis, which can be obtained only by molecular methods, seems to be crucial in patients management. Hence, future directions should focus on revealing the molecular cause in individuals with HME and negative *EXT1* and *EXT2* mutational screening and better molecular diagnostics of pediatric patients. In addition, the second reachable goal should aim at early recognition of HMEs malignant transformation and its better understanding at the molecular and cellular level. Achieving the above will probably reveal new treatment options or therapeutic targets in both HMEs and CHS.

## References

[B1] Abdolrazaghi H. Riyahi A. Taghavi M. Farshidmehr P. Mohammadbeigi A. (2018). Concomitant Neurogenic and Vascular Thoracic Outlet Syndrome Due to Multiple Exostoses. Ann. Card. Anaesth. 21, 71–73. 10.4103/aca.ACA_119_17 29336398 PMC5791495

[B2] Ahn J. Lüdecke H.-J. Lindow S. Horton W. A. Lee B. Wagner M. J. (1995). Cloning of the Putative Tumour Suppressor Gene for Hereditary Multiple Exostoses (EXT1). Nat. Genet. 11, 137–143. 10.1038/ng1095-137 7550340

[B3] Al-Zayed Z. Al-Rijjal R. A. Al-Ghofaili L. BinEssa H. A. Pant R. Alrabiah A. (2021). Mutation Spectrum of EXT1 and EXT2 in the Saudi Patients with Hereditary Multiple Exostoses. Orphanet J. Rare Dis. 16, 100. 10.1186/s13023-021-01738-z 33632255 PMC7905910

[B4] Alabdullrahman L. W. Byerly D. W. (2021). Osteochondroma. Treasure Island (FL): StatPearls Publishing. 31335016

[B5] Alvarez C. Tredwell S. De Vera M. Hayden M. (2006). The Genotype-Phenotype Correlation of Hereditary Multiple Exostoses. Clin. Genet. 70, 122–130. 10.1111/j.1399-0004.2006.00653.x 16879194

[B6] Anower-E-Khuda M. F. Matsumoto K. Habuchi H. Morita H. Yokochi T. Shimizu K. (2013). Glycosaminoglycans in the Blood of Hereditary Multiple Exostoses Patients: Half Reduction of Heparan Sulfate to Chondroitin Sulfate Ratio and the Possible Diagnostic Application. Glycobiology 23, 865–876. 10.1093/glycob/cwt024 23514715

[B7] Beltrami G. Ristori G. Scoccianti G. Tamburini A. Capanna R. (2016). Hereditary Multiple Exostoses: A Review of Clinical Appearance and Metabolic Pattern. Clin. Cases Miner Bone Metab. 13, 110–118. 10.11138/ccmbm/2016.13.2.110 27920806 PMC5119707

[B8] Beyens A. Meensel K. Pottie L. Rycke R. Bruyne M. Baeke F. (2019). Defining the Clinical, Molecular and Ultrastructural Characteristics in Occipital Horn Syndrome: Two New Cases and Review of the Literature. Genes 10, 528. 10.3390/genes10070528 PMC6678539 31336972

[B9] Billings P. C. Pacifici M. (2015). Interactions of Signaling Proteins, Growth Factors and Other Proteins with Heparan Sulfate: Mechanisms and Mysteries. Connect. Tissue Res. 56, 272–280. 10.3109/03008207.2015.1045066 26076122 PMC4785798

[B10] Bovée J. V. M. G. Hogendoorn P. C. W. Wunder J. S. Alman B. A. (2010). Cartilage Tumours and Bone Development: Molecular Pathology and Possible Therapeutic Targets. Nat. Rev. Cancer 10, 481–488. 10.1038/nrc2869 20535132

[B11] Bovée J. V. (2008). Multiple Osteochondromas. Orphanet J. Rare Dis. 3, 3. 10.1186/1750-1172-3-3 18271966 PMC2276198

[B12] Busse-Wicher M. Wicher K. B. Kusche-Gullberg M. (2014). The Extostosin Family: Proteins with many Functions. Matrix Biol. 35, 25–33. 10.1016/j.matbio.2013.10.001 24128412

[B13] Charifa A. Jamil R. T. Zhang X. (2021). Gardner Syndrome. Treasure Island (FL): StatPearls Publishing. 29493967

[B14] Chhina H. Davis J. C. Alvarez C. M. (2012). Health-related Quality of Life in People with Hereditary Multiple Exostoses. J. Pediatr. Orthop. 32, 210–214. 10.1097/BPO.0b013e31823ee31c 22327458

[B15] Clement N. Porter D. (2014). Hereditary Multiple Exostoses: Anatomical Distribution and burden of Exostoses Is Dependent upon Genotype and Gender. Scott. Med. J. 59, 35–44. 10.1177/0036933013518150 24413927

[B16] Cowles R. A. Rowe D. H. Arkovitz M. S. (2005). Hereditary Multiple Exostoses of the Ribs: an Unusual Cause of Hemothorax and Pericardial Effusion. J. Pediatr. Surg. 40, 1197–1200. 10.1016/j.jpedsurg.2005.03.064 16034772

[B17] Czajka C. M. DiCaprio M. R. (2015). What Is the Proportion of Patients with Multiple Hereditary Exostoses Who Undergo Malignant Degeneration? Clin. Orthop. Relat. Res. 473, 2355–2361. 10.1007/s11999-015-4134-z 25582066 PMC4457763

[B18] D'Arienzo A. Andreani L. Sacchetti F. Colangeli S. Capanna R. (2019). Hereditary Multiple Exostoses: Current Insights. Orthop. Res. Rev. 11, 199–211. 10.2147/ORR.S183979 31853203 PMC6916679

[B19] Darilek S. Wicklund C. Novy D. Scott A. Gambello M. Johnston D. (2005). Hereditary Multiple Exostosis and Pain. J. Pediatr. Orthop. 25, 369–376. 10.1097/01.bpo.0000150813.18673.ad 15832158

[B20] Fei L. Ngoh C. Porter D. E. (2018). Chondrosarcoma Transformation in Hereditary Multiple Exostoses: A Systematic Review and Clinical and Cost-Effectiveness of a Proposed Screening Model. J. Bone Oncol. 13, 114–122. 10.1016/j.jbo.2018.09.011 30591865 PMC6303411

[B21] Francannet C. Cohen-Tanugi A. Le Merrer M. Munnich A. Bonaventure J. Legeai-Mallet L. (2001). Genotype-Phenotype Correlation in Hereditary Multiple Exostoses. J. Med. Genet. 38, 430–434. 10.1136/jmg.38.7.430 11432960 PMC1757186

[B22] Fusco C. Nardella G. Fischetto R. Copetti M. Petracca A. Annunziata F. (2019). Mutational Spectrum and Clinical Signatures in 114 Families with Hereditary Multiple Osteochondromas: Insights into Molecular Properties of Selected Exostosin Variants. Hum. Mol. Genet. 28, 2133–2142. 10.1093/hmg/ddz046 30806661

[B23] Goud A. L. de Lange J. Scholtes V. A. B. Bulstra S. K. Ham S. J. (2012). Pain, Physical and Social Functioning, and Quality of Life in Individuals with Multiple Hereditary Exostoses in the Netherlands. J. Bone Jt. Surg. Am. 94, 1013–1020. 10.2106/JBJS.K.00406 22637207

[B24] Gulati A. Mittal A. Singal R. Gupta S. Garg V. (2013). A Unique Case of Cervical Osteochondroma Causing Dysphagia. Kulak Burun Bogaz Ihtis. Derg. 23, 246–248. 10.5606/kbbihtisas.2013.41736 23834138

[B25] Guo X.-L. Deng Y. Liu H.-G. (2014). Clinical Characteristics of Hereditary Multiple Exostoses: a Retrospective Study of mainland Chinese Cases in Recent 23 Years. J. Huazhong Univ. Sci. Technolog. Med. Sci. 34, 42–50. 10.1007/s11596-014-1230-3 24496678

[B26] Hecht J. T. Hogue D. Wang Y. Blanton S. H. Wagner M. Strong L. C. (1997). Hereditary Multiple Exostoses (EXT): Mutational Studies of Familial EXT1 Cases and EXT-Associated Malignancies. Am. J. Hum. Genet. 60, 80–86. 8981950 PMC1712567

[B27] Hennekam R. C. (1991). Hereditary Multiple Exostoses. J. Med. Genet. 28, 262–266. 10.1136/jmg.28.4.262 1856833 PMC1016830

[B28] Herget G. W. Kontny U. Saueressig U. Baumhoer D. Hauschild O. Elger T. (2013). Osteochondrom und Multiple Osteochondrome. Radiologe 53, 1125–1136. 10.1007/s00117-013-2571-9 24129968

[B29] Heuzé Y. Holmes G. Peter I. Richtsmeier J. T. Jabs E. W. (2014). Closing the Gap: Genetic and Genomic Continuum from Syndromic to Nonsyndromic Craniosynostoses. Curr. Genet. Med. Rep. 2, 135–145. 10.1007/s40142-014-0042-x 26146596 PMC4489147

[B30] Hoffman L. M. Weston A. D. Underhill T. M. (2003). Molecular Mechanisms Regulating Chondroblast Differentiation. The J. Bone Jt. Surgery-American Volume 85, 124–132. 10.2106/00004623-200300002-00017 12721355

[B31] Huegel J. Enomoto-Iwamoto M. Sgariglia F. Koyama E. Pacifici M. (2015). Heparanase Stimulates Chondrogenesis and Is Up-Regulated in Human Ectopic Cartilage. Am. J. Pathol. 185, 1676–1685. 10.1016/j.ajpath.2015.02.014 25863260 PMC4450318

[B32] Huegel J. Sgariglia F. Enomoto-Iwamoto M. Koyama E. Dormans J. P. Pacifici M. (2013). Heparan Sulfate in Skeletal Development, Growth, and Pathology: The Case of Hereditary Multiple Exostoses. Dev. Dyn. 242, 1021–1032. 10.1002/dvdy.24010 23821404 PMC4007065

[B33] Inubushi T. Lemire I. Irie F. Yamaguchi Y. (2018). Palovarotene Inhibits Osteochondroma Formation in a Mouse Model of Multiple Hereditary Exostoses. J. Bone Miner Res. 33, 658–666. 10.1002/jbmr.3341 29120519 PMC5895492

[B34] Ishimaru D. Gotoh M. Takayama S. Kosaki R. Matsumoto Y. Narimatsu H. (2016). Large-Scale Mutational Analysis in the EXT1 and EXT2 Genes for Japanese Patients with Multiple Osteochondromas. BMC Genet. 17, 52. 10.1186/s12863-016-0359-4 26961984 PMC4784393

[B35] Jäger M. Westhoff B. Portier S. Leube B. Hardt K. Royer-Pokora B. (2007). Clinical Outcome and Genotype in Patients with Hereditary Multiple Exostoses. J. Orthop. Res. 25, 1541–1551. 10.1002/jor.20479 17676624

[B36] Jamsheer A. Socha M. Sowińska-Seidler A. Telega K. Trzeciak T. Latos-Bieleńska A. (2014). Mutational Screening of EXT1 and EXT2 Genes in Polish Patients with Hereditary Multiple Exostoses. J. Appl. Genet. 55, 183–188. 10.1007/s13353-014-0195-z 24532482 PMC3990859

[B37] Jones K. B. (2011). Glycobiology and the Growth Plate. J. Pediatr. Orthop. 31, 577–586. 10.1097/BPO.0b013e31821c7738 21654469 PMC3111916

[B38] Jones K. B. Pacifici M. Hilton M. J. (2014). Multiple Hereditary Exostoses (MHE): Elucidating the Pathogenesis of a Rare Skeletal Disorder through Interdisciplinary Research. Connect. Tissue Res. 55, 80–88. 10.3109/03008207.2013.867957 24409815

[B39] Jurik A. G. Jørgensen P. H. Mortensen M. M. (2020). Whole-Body MRI in Assessing Malignant Transformation in Multiple Hereditary Exostoses and Enchondromatosis: Audit Results and Literature Review. Skeletal Radiol. 49, 115–124. 10.1007/s00256-019-03268-z 31273432

[B40] Jurik A. G. (2020). Multiple Hereditary Exostoses and Enchondromatosis. Best Pract. Res. Clin. Rheumatol. 34, 101505. 10.1016/j.berh.2020.101505 32253147

[B41] Kok H. K. Fitzgerald L. Campbell N. Lyburn I. D. Munk P. L. Buckley O. (2013). Multimodality Imaging Features of Hereditary Multiple Exostoses. Bjr 86, 20130398. 10.1259/bjr.20130398 24004486 PMC3798337

[B42] Le Merrer M. Legeai-Mallet L. Jeannin P. M. Horsthemke B. Schlnzel A. Plauchu H. (1994). A Gene for Hereditary Multiple Exostoses Maps to Chromosome 19p. Hum. Mol. Genet. 3, 717–722. 10.1093/hmg/3.5.717 8081357

[B43] Li J.-P. Kusche-Gullberg M. (2016). Heparan Sulfate: Biosynthesis, Structure, and Function. Int. Rev. Cel. Mol. Biol. 325, 215–273. 10.1016/bs.ircmb.2016.02.009 27241222

[B44] Li Y. Wang J. Tang J. Wang Z. Han B. Li N. (2018). Heterogeneous Spectrum of EXT Gene Mutations in Chinese Patients with Hereditary Multiple Osteochondromas. Medicine (Baltimore) 97, e12855. 10.1097/MD.0000000000012855 30334991 PMC6211902

[B45] Liang C. Wang Y. J. Wei Y. X. Dong Y. Zhang Z. C. (2020). Identification of Novel EXT Mutations in Patients with Hereditary Multiple Exostoses Using Whole‐Exome Sequencing. Orthop. Surg. 12, 990–996. 10.1111/os.12660 32293802 PMC7307237

[B46] Lonie L. Porter D. E. Fraser M. Cole T. Wise C. Yates L. (2006). Determination of the Mutation Spectrum of the EXT1/EXT2 genes in British Caucasian Patients with Multiple Osteochondromas, and Exclusion of Six Candidate Genes in EXT negative Cases. Hum. Mutat. 27, 1160. 10.1002/humu.9467 17041877

[B47] Lopes C. C. Dietrich C. P. Nader H. B. (2006). Specific Structural Features of Syndecans and Heparan Sulfate Chains Are Needed for Cell Signaling. Braz. J. Med. Biol. Res. 39, 157–167. 10.1590/s0100-879x2006000200001 16470302

[B48] Maas S. Shaw A. Bikker H. Hennekam R. C. M. (1993). Trichorhinophalangeal Syndrome. Editors Adam M. P. Ardinger H. H. Pagon R. A. Wallace S. E. Bean L. J. H. Mirzaa G. (Seattle (WA): University of Washington). 28426188

[B49] Majumdar A. K. (1994). Hereditary Multiple Exostoses. J. Indian Med. Assoc. 92 (59), 59–63. 8071561

[B50] Matsumoto Y. Matsumoto K. Harimaya K. Okada S. Doi T. Iwamoto Y. (2015). Scoliosis in Patients with Multiple Hereditary Exostoses. Eur. Spine J. 24, 1568–1573. 10.1007/s00586-015-3883-4 25794701

[B51] McFarlane J. Knight T. Sinha A. Cole T. Kiely N. Freeman R. (2016). Exostoses, Enchondromatosis and Metachondromatosis; Diagnosis and Management. Acta Orthop. Belg. 82, 102–105. 26984661

[B52] Medek K. Zeman J. Honzík T. Hansíková H. Švecová Š. Beránková K. (2017). Hereditary Multiple Exostoses: Clinical, Molecular and Radiologic Survey in 9 Families. Prague Med. Rep. 118, 87–94. 10.14712/23362936.2017.8 28922105

[B53] Mikami T. Kitagawa H. (2013). Biosynthesis and Function of Chondroitin Sulfate. Biochim. Biophys. Acta (Bba) - Gen. Subjects 1830, 4719–4733. 10.1016/j.bbagen.2013.06.006 23774590

[B54] Mordenti M. Ferrari E. Pedrini E. Fabbri N. Campanacci L. Muselli M. (2013). Validation of a New Multiple Osteochondromas Classification through Switching Neural Networks. Am. J. Med. Genet. 161, 556–560. 10.1002/ajmg.a.35819 23401177

[B55] Mundy C. Bello A. Sgariglia F. Koyama E. Pacifici M. (2016). HhAntag, a Hedgehog Signaling Antagonist, Suppresses Chondrogenesis and Modulates Canonical and Non-Canonical BMP Signaling. J. Cel. Physiol. 231, 1033–1044. 10.1002/jcp.25192 26363135

[B56] Murphey M. D. Choi J. J. Kransdorf M. J. Flemming D. J. Gannon F. H. (2000). Imaging of Osteochondroma: Variants and Complications with Radiologic-Pathologic Correlation. Radiographics 20, 1407–1434. 10.1148/radiographics.20.5.g00se171407 10992031

[B57] Pacifici M. (2018). Hereditary Multiple Exostoses: Are There New Plausible Treatment Strategies? Expert Opin. Orphan Drugs 6, 385–391. 10.1080/21678707.2018.1483232 31448184 PMC6707746

[B58] Pacifici M. (2017). Hereditary Multiple Exostoses: New Insights into Pathogenesis, Clinical Complications, and Potential Treatments. Curr. Osteoporos. Rep. 15, 142–152. 10.1007/s11914-017-0355-2 28466453 PMC5510481

[B59] Pedrini E. Jennes I. Tremosini M. Milanesi A. Mordenti M. Parra A. (2011). Genotype-Phenotype Correlation Study in 529 Patients with Multiple Hereditary Exostoses: Identification of “Protective” and “Risk” Factors. J. Bone Jt. Surgery-American Volume 93, 2294–2302. 10.2106/JBJS.J.00949 22258776

[B60] Phan A. Q. Pacifici M. Esko J. D. (2018). Advances in the Pathogenesis and Possible Treatments for Multiple Hereditary Exostoses from the 2016 International MHE Conference. Connect. Tissue Res. 59, 85–98. 10.1080/03008207.2017.1394295 29099240 PMC7604901

[B61] Pignolo R. J. Shore E. M. Kaplan F. S. (2013). Fibrodysplasia Ossificans Progressiva: Diagnosis, Management, and Therapeutic Horizons. Pediatr. Endocrinol. Rev. 10 (Suppl. 2), 437–448. 23858627 PMC3995352

[B62] Ravindran R. Jordan S. Bush A. (2015). An Extra Piece of Grey. Thorax 70, 705–706. 10.1136/thoraxjnl-2015-207061 25935166

[B63] Ryckx A. Somers J. F. Allaert L. (2013). Hereditary Multiple Exostosis. Acta Orthop. Belg. 79, 597–607. 24563962

[B64] Sabir A. H. Cole T. (2019). The Evolving Therapeutic Landscape of Genetic Skeletal Disorders. Orphanet J. Rare Dis. 14, 300. 10.1186/s13023-019-1222-2 31888683 PMC6937740

[B65] Santos S. C. L. Rizzo I. M. P. O. Takata R. I. Speck-Martins C. E. Brum J. M. Sollaci C. (2018). Analysis of Mutations in EXT1 and EXT2 in Brazilian Patients with Multiple Osteochondromas. Mol. Genet. Genomic Med. 6, 382–392. 10.1002/mgg3.382 29529714 PMC6014457

[B66] Sarrión P. Sangorrin A. Urreizti R. Delgado A. Artuch R. Martorell L. (2013). Mutations in the EXT1 and EXT2 Genes in Spanish Patients with Multiple Osteochondromas. Sci. Rep. 3, 1346. 10.1038/srep01346 23439489 PMC3581825

[B67] Schmale G. A. Conrad E. U. 3rd Raskind W. H. (1994). The Natural History of Hereditary Multiple Exostoses. J. Bone Jt. Surg. 76, 986–992. 10.2106/00004623-199407000-00005 8027127

[B68] Seki H. Kubota T. Ikegawa S. Haga N. Fujioka F. Ohzeki S. (2001). Mutation Frequencies ofEXT1 andEXT2 in 43 Japanese Families with Hereditary Multiple Exostoses. Am. J. Med. Genet. 99, 59–62. 10.1002/1096-8628(20010215)99:1<59:aid-ajmg1115>3.0.co;2-z 11170095

[B69] Shimono K. Tung W.-E. Macolino C. Chi A. H.-T. Didizian J. H. Mundy C. (2011). Potent Inhibition of Heterotopic Ossification by Nuclear Retinoic Acid Receptor-γ Agonists. Nat. Med. 17, 454–460. 10.1038/nm.2334 21460849 PMC3073031

[B70] Sinha S. Mundy C. Bechtold T. Sgariglia F. Ibrahim M. M. Billings P. C. (2017). Unsuspected Osteochondroma-like Outgrowths in the Cranial Base of Hereditary Multiple Exostoses Patients and Modeling and Treatment with a BMP Antagonist in Mice. Plos Genet. 13, e1006742. 10.1371/journal.pgen.1006742 28445472 PMC5425227

[B71] Staal H. M. Goud A. L. van der Woude H.-J. Witlox M. A. Ham S. J. Robben S. G. F. (2015). Skeletal Maturity of Children with Multiple Osteochondromas: Is Diminished Stature Due to a Systemic Influence? J. Child. Orthop. 9, 397–402. 10.1007/s11832-015-0680-x 26320759 PMC4619368

[B72] Stickens D. Clines G. Burbee D. Ramos P. Thomas S. Hogue D. (1996). The EXT2 Multiple Exostoses Gene Defines a Family of Putative Tumour Suppressor Genes. Nat. Genet. 14, 25–32. 10.1038/ng0996-25 8782816

[B73] Stieber J. R. Dormans J. P. (2005). Manifestations of Hereditary Multiple Exostoses. J. Am. Acad. Orthopaedic Surgeons 13, 110–120. 10.5435/00124635-200503000-00004 15850368

[B74] Suster D. Hung Y. P. Nielsen G. P. (2020). Differential Diagnosis of Cartilaginous Lesions of Bone. Arch. Pathol. Lab. Med. 144, 71–82. 10.5858/arpa.2019-0441-RA 31877083

[B75] Trajkova S. Di Gregorio E. Ferrero G. B. Carli D. Pavinato L. Delplancq G. (2020). New Insights into Potocki-Shaffer Syndrome: Report of Two Novel Cases and Literature Review. Brain Sci. 10, 788. 10.3390/brainsci10110788 PMC7693731 33126574

[B76] Trebicz-Geffen M. Robinson D. Evron Z. Glaser T. Fridkin M. Kollander Y. (2008). The Molecular and Cellular Basis of Exostosis Formation in Hereditary Multiple Exostoses. Int. J. Exp. Pathol. 89, 321–331. 10.1111/j.1365-2613.2008.00589.x 18452536 PMC2613984

[B77] Van den Wyngaert T. Elvas F. De Schepper S. Kennedy J. A. Israel O. (2020). SPECT/CT: Standing on the Shoulders of Giants, it Is Time to Reach for the Sky!. J. Nucl. Med. 61, 1284–1291. 10.2967/jnumed.119.236943 32620702

[B78] Vanhoenacker F. M. Van Hul W. Wuyts W. Willems P. J. De Schepper A. M. (2001). Hereditary Multiple Exostoses: from Genetics to Clinical Syndrome and Complications. Eur. J. Radiol. 40, 208–217. 10.1016/s0720-048x(01)00401-6 11731209

[B79] Vu C. L. Lindberg A. W. Bompadre V. White K. K. Bauer J. M. (2020). Prospective Spine at Risk Program for Prevalence of Intracanal Spine Lesions in Pediatric Hereditary Multiple Osteochondromas. Spine Deform. 8, 1069–1074. 10.1007/s43390-020-00130-4 32367382

[B80] Wang Y.-G. Xie P. Wang Y.-G. Li X.-D. Zhang T.-G. Liu Z.-Y. (2014). All-Trans-Retinoid Acid (ATRA) Suppresses Chondrogenesis of Rat Primary Hind Limb Bud Mesenchymal Cells by Downregulating P63 and Cartilage-Specific Molecules. Environ. Toxicol. Pharmacol. 38, 460–468. 10.1016/j.etap.2014.07.008 25136779

[B81] Wang Y. Zhong L. Xu Y. Ding L. Ji Y. Schutz S. (2020). EXT1 and EXT2 Variants in 22 Chinese Families with Multiple Osteochondromas: Seven New Variants and Potentiation of Preimplantation Genetic Testing and Prenatal Diagnosis. Front. Genet. 11, 607838. 10.3389/fgene.2020.607838 33414810 PMC7783290

[B82] Wang Z. Zou Y. Chen Y. Chen Y. (2018). Multiple Unexpected Lesions of Metachondromatosis Detected by Technetium-99m Methylene Diphosphonate SPECT/CT. Medicine (Baltimore) 97, e0512. 10.1097/MD.0000000000010512 29703018 PMC5944487

[B83] Weston A. D. Chandraratna R. A. S. Torchia J. Underhill T. M. (2002). Requirement for RAR-Mediated Gene Repression in Skeletal Progenitor Differentiation. J. Cel. Biol. 158, 39–51. 10.1083/jcb.200112029 PMC2173026 12105181

[B84] Wuyts W. Ramlakhan S. Van Hul W. Hecht J. T. van den Ouweland A. M. Raskind W. H. (1995). Refinement of the Multiple Exostoses Locus (EXT2) to a 3-cM Interval on Chromosome 11. Am. J. Hum. Genet. 57, 382–387. 7668264 PMC1801560

[B85] Wuyts W. Schmale G. A. Chansky H. A. Raskind W. H. (1993). Hereditary Multiple Osteochondromas. Editors Adam M. P. Ardinger H. H. Pagon R. A. Wallace S. E. Bean L. J. H. Mirzaa G.. Seattle, WA: University of Washington. 20301413

[B86] Xu L. Xia J. Jiang H. Zhou J. Li H. Wang D. (1999). Mutation Analysis of Hereditary Multiple Exostoses in the Chinese. Hum. Genet. 105, 45–50. 10.1007/s004399900058 10480354

[B87] Yoon B. S. Pogue R. Ovchinnikov D. A. Yoshii I. Mishina Y. Behringer R. R. (2006). BMPs Regulate Multiple Aspects of Growth-Plate Chondrogenesis through Opposing Actions on FGF Pathways. Development 133, 4667–4678. 10.1242/dev.02680 17065231

